# Constructing a risk screen for attention difficulty in U.S. adults using six machine learning methods

**DOI:** 10.3389/frai.2025.1704576

**Published:** 2026-01-12

**Authors:** Ying Song, Yansun Sun, Zedan Guo, Li Yi

**Affiliations:** 1 Department of Neurology, Peking University Shenzhen Hospital, Shenzhen, China; 2 Department of Geriatrics, Peking University Shenzhen Hospital, Shenzhen, China

**Keywords:** machine learning, NHANES, concentration difficulty, neuropsychiatric disorders, logistic regression

## Abstract

**Background:**

Concentration difficulty is recognized as a hallmark of various neurologic and neuropsychiatric disorders. However, an accurate estimation of epidemiological risk factors for concentration difficulty remains severely limited.

**Aims:**

The study aimed to develop an interpretable machine-learning (ML) model to predict risk factors of concentration difficulty among adults in the United States.

**Methods:**

A total of 9,971 participants were included from the 2015–2016 cycle of the National Health and Nutrition Examination Survey (NHANES). Six ML algorithms, including Logistic Regression, ExtraTrees classifier, Bagging, Gradient Boosting, Extreme Gradient Boosting (XGBoost), and Random Forest (RF), were applied in this study. Model performance was evaluated using the area under the receiver operating characteristic curve (AUC), accuracy, precision, specificity, decision curve analysis (DCA), and calibration plots. Finally, a nomogram was constructed based on the best performing model.

**Results:**

Of these, 2,146 participants aged 20 years and older were analyzed. Logistic regression exhibited the best clinical predictive value in both internal and external validation sets, with AUCs of 0.881 and 0.818, respectively. The DCA curve revealed that logistic regression exhibited the greatest net benefits in the internal cohort, whereas the RF model provided the largest net benefits in the external cohort (threshold: 0.2–0.3).

**Conclusion:**

Logistic regression exhibited the highest clinical value in predicting concentration difficulty. These findings provide valuable insights for the recognition, management, and effective interference strategies for concentration difficulty.

## Introduction

Concentration difficulty is a common complaint among psychopathological patients as well as a hallmark of neurologic and neuropsychiatric disorders, including anxiety, major depressive disorder (MDD), schizophrenia, post-traumatic stress disorder (PTSD), and Alzheimer’s disease (AD) (Hallion et al., 2018; Keller et al., 2019; Khanna et al., 2017; Luck et al., 2019). For example, patients with anxiety disorders exhibit a higher prevalence of concentration issues across various age groups (Rodrigues et al., 2019). Individuals with schizophrenia are characterized by impaired concentration and altered processing speed (Egeland et al., 2003). Among patients with mild-to-moderate AD, concentration impairments are observed in more than 80% (Gilmour et al., 2019). Concentration difficulties are also frequently reported in patients with post-stroke aphasia (Schumacher et al., 2019). Recognition and management remain challenging, as no specific biochemical or imaging abnormalities are available, particularly in patients with overlapping etiologies or uncertain causes (Hallion et al., 2018). Therefore, the best treatment procedures are often missed, leading to poor outcomes in psychosocial and occupational domains and adding to the overall burden on society worldwide (Bornert and Bouret, 2021).

Large-scale national surveys were conducted to identify prevalence and risk factors pertaining to concentration difficulties. Existing models of attention describe the association between risk factors and concentration difficulties, contributing to symptom evaluations (Cao et al., 2023; Gong et al., 2022). However, systematic estimation of risk prediction model for attention difficulty remains insufficient. Traditionally, the interaction between these risk factors and their clinical values has been limited (Fardell et al., 2023; Epstein and Kumra, 2014). In addition, a majority of existing risk prediction models for concentration difficulties were limited to children and teenagers, including attention-deficit/hyperactivity disorder (ADHD), which may not apply to adult patients. Thus, it is of great clinical significance to establish precise risk screen models for concentration difficulties and to optimize the management of high-risk adult individuals.

Artificial intelligence (AI) is increasingly applied to identify early indications of diseases. As a key branch of AI, machine learning (ML) algorithms can analyze diverse features, thereby improving diagnostic accuracy (Alber et al., 2019). ML applications have achieved major breakthroughs in various medical fields. For example, ML model improves the prediction of heart failure, stroke, cancer, and psychiatric disorders (Chen et al., 2023; Li et al., 2022; Huang et al., 2020; Elemento et al., 2021; Dwyer et al., 2018). These findings suggest that ML could be a powerful technique for enhancing diagnostic accuracy, risk prediction, and intervention strategies.

**Figure 1:**
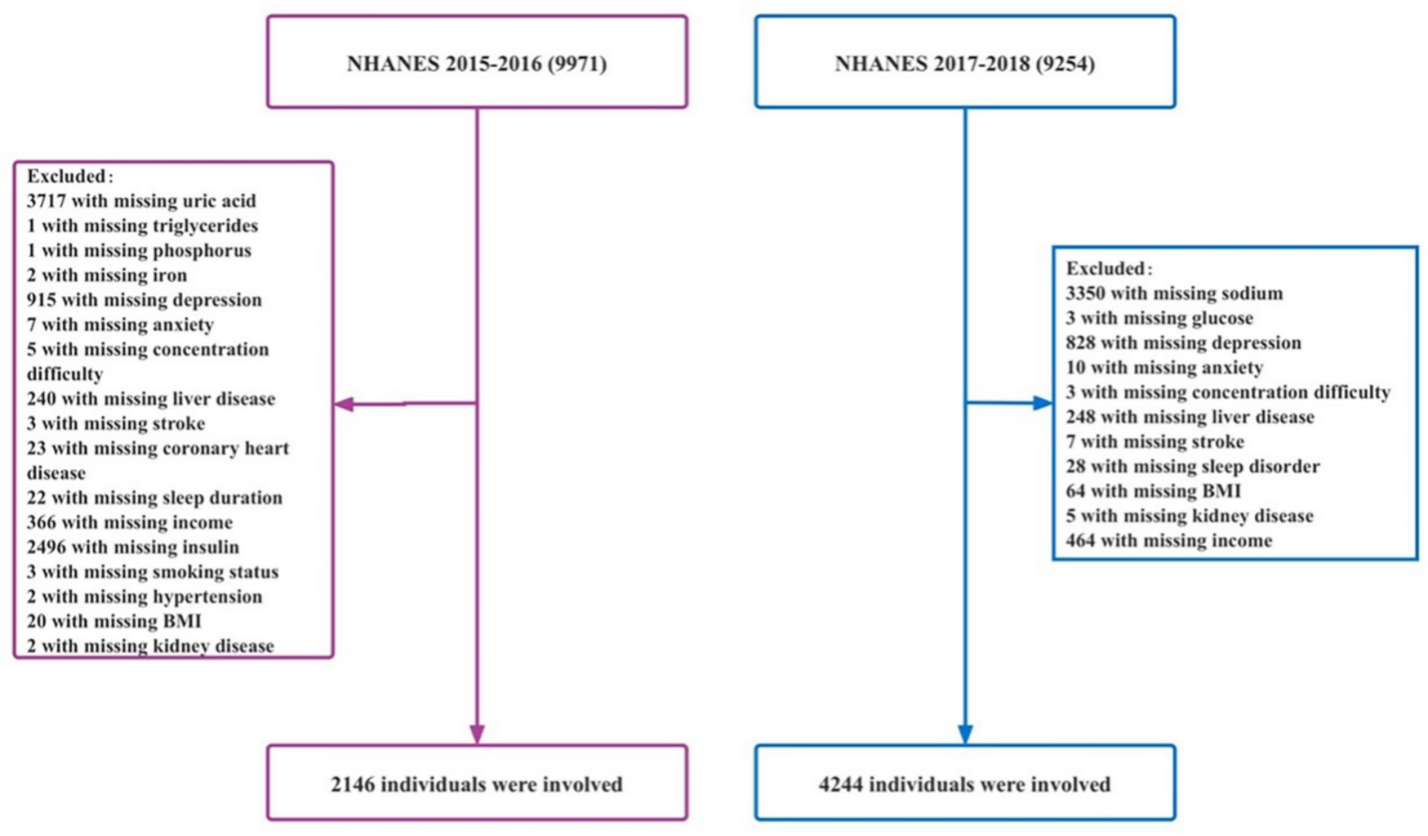
Flowchart of the study population. BMI, body mass index.

To our knowledge, few studies have concentrated on the prediction of risk factors in concentration difficulty using ML approaches, especially in adult patients. This study is aimed at developing and validating the risks associated with concentration difficulties when using six ML models based on the NHANES database. The inclusion and exclusion criteria of this study are shown in Figure 1.

## Materials and methods

### Study design and participants

According to a nationally representative database, NHANES is sponsored by the Centers for Disease Control and Prevention (CDC) and aims to assess the health and nutrition status of both adults and children in the United States (Cheng et al., 2023). The survey samples the U.S. civilian population using a stratified, multistage probability design and collects nationally representative data based on demographic data, diet, physical examination, laboratory measures, and questionnaires (Song et al., 2022).

A total of 9,971 adults from the 2015–2016 NHANES cycles were included in the study, and demographic, physical examination, laboratory, and questionnaire data were analyzed. A total of 24 predictors related to concentration difficulty were considered. After excluding individuals with missing data on uric acid (*N* = 3,717), triglycerides (*N* = 1), phosphorus (*N* = 1), iron (*N* = 2), depression (*N* = 915), anxiety (*N* = 7), concentration difficulty (*N* = 5), as well as patients who had a history of liver disease (*N* = 240), stroke (*N* = 3), coronary heart disease (*N* = 23), sleep duration (*N* = 22), income criteria (*N* = 366), insulin (*N* = 2,496), smoking status (*N* = 3), hypertension (*N* = 2), body mass index (BMI, *N* = 20), and kidney disease (*N* = 2), the final sample consisted of 2,146 participants.

### Concentration difficulties

The 2015–2018 NHANES survey assessed attention difficulties using a disability questionnaire supplied in a Mobile Examination Center. The questionnaire collected respondent-level interview data on serious difficulties associated with hearing, seeing, concentrating, walking, dressing, and running errands. Its development involved extensive input from federal agencies, consultants, and experts from external research community. The primary outcome for this analysis was based on responses (yes or no) to the questions: Do you have serious difficulty concentrating? (Fardell et al., 2023).

### Other covariates

Known risk factors, along with demographic and disease characteristics of clinical importance, were selected as candidate variables for the prediction model (Kim et al., 2016). In this study, demographic factors include age (20–80 years), sex (male and female), and income criteria (Zhou et al., 2024). Lifestyle variables comprised BMI, sleep duration, and smoking status (subjects having smoked fewer than 100 cigarettes in one’s lifetime or not) (Aronow and Frishman, 2018; Yakushiji et al., 2018; Aaron and Hughes, 2007; Deierlein et al., 2022; Huang et al., 2021). Health-related variables included in the questionnaire were hypertension, coronary heart disease, stroke, cancer, liver disease, kidney disease, anxiety, and depression. Laboratory data consisted of concentrations of calcium, cholesterol, chloride, glucose, insulin, iron, potassium, sodium, phosphorus, triglycerides, and uric acid in blood.

### Machine learning model development

LASSO regression is a powerful technique for creating parsimonious models while mitigating issues related to overfitting (Tsur et al., 2020). In this study, the LASSO regression model was constructed using the optimal alpha parameter to select variables most strongly associated with concentration difficulties and to calculate the importance values for each feature (Cai et al., 2023). During the elimination process, 5-fold cross-validation was applied to optimize the hyperparameters for each model. For feature selection, the top 14 meaningful variables selected by LASSO regression were incorporated into ML models for prediction.

The dataset was randomly partitioned into a training set (80%, *N* = 1716) and a testing set (20%, *N* = 430). Feature selection and hyperparameter tuning were conducted on the training set to develop models for each ML algorithm, and the trained models were applied on the testing set for evaluation. A grid search with 5-fold cross-validation was used to optimize the hyperparameters of each algorithm.

Six ML algorithms were conducted. Logistic regression, a generalized linear model, is commonly used for solving binary problems. In this study, logistic regression with L2 regularization was conducted to reduce the effects of feature correlation and prevent overfitting. Bagging is an ensemble learning algorithm that integrates bootstrapping and aggregation techniques (Mehrbakhsh et al., 2024). Gradient boosting can effectively reduce bias and variance by optimizing the loss function during the learning process (Wijaya et al., 2024). RF employs bootstrap resampling to repeatedly and randomly select B samples from the training sample set, in which *N* is the training set, and the remaining samples serve as the test set (Zhong et al., 2023). The ExtraTrees classifier adds innovative algorithmic steps based on the traditional algorithm of Decision Tree (DT) and provides very strong additional randomness to suppress overfitting (Lin et al., 2024). As an optimized Gradient Boosting algorithm, the Extreme Gradient Boost (XGBoost) avoids the overfitting issue by incorporating a regularization component in the objective function and approximates the loss function using the second-order Taylor expansion (Bi et al., 2020).

**Figure 2:**
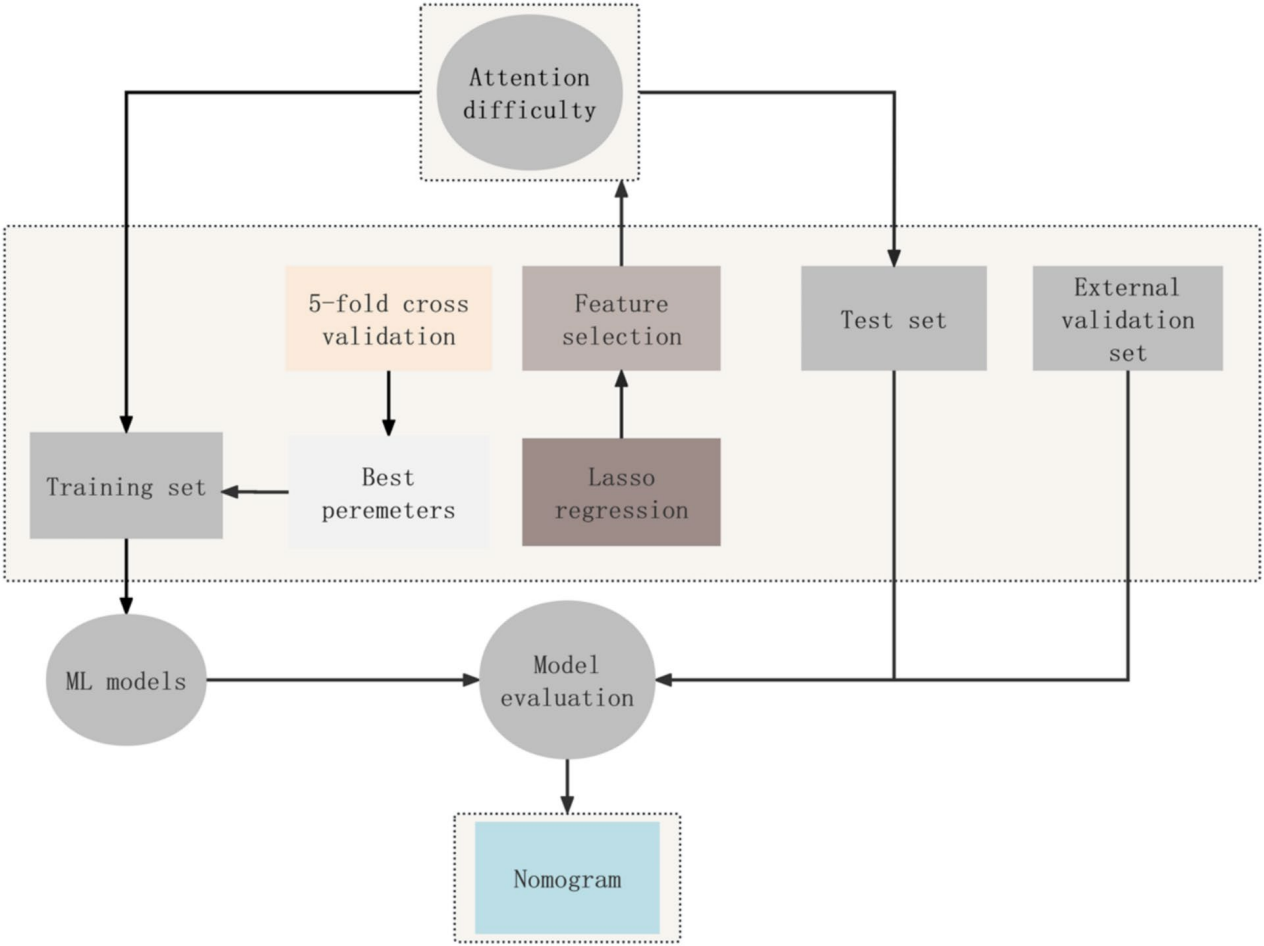
Study design to construct machine learning models to predict the risk of concentration difficulty. ML, machine learning.

Figure 2 shows a diagram of the concentration difficulty risk prediction framework.

### Evaluation of a machine learning model

The performance of the prediction model was evaluated through confusion matrix, accuracy (the percentage of positive samples to all samples), AUC (area under the curve), precision (the correct proportion of the predicted positive samples), specificity (the proportion of predicted negative samples to negative samples), F1 (the harmonic means of precision and recall), and recall (the proportion of predicted positive samples to all positive samples) (Kumar et al., 2022; Liu et al., 2022). In addition, DCA was performed to evaluate whether a model has utility in supporting clinical decisions by calculating the net benefit over a range of threshold probabilities (Raita et al., 2019). The vertical axis represents the standardized net benefit, while the horizontal axes depict the risk threshold. The greater standardized net benefit (reflected by a larger area under the curve) indicates that the model’s clinical decision is more advantageous (Zhang et al., 2024). Moreover, the calibration curves were used to assess the model calibration between the predicted probabilities and the actual probabilities (Gu et al., 2024; Xiang et al., 2024). In addition, to further validate the performance of the prediction model, participants from the 2017–2018 NHANES cycle were included as an external validation set. The primary outcomes used to assess the accuracy and clinical efficacy of the model in this external validation cohort were the AUC, DCA, and calibration curves.

### Development of the nomogram

The nomogram functions by integrating various prognostic and determinant data is used to estimate the individual probability of a clinical occurrence (Balachandran et al., 2015). The nomogram links each variable with its corresponding score, and the cumulative sum of all the variable scores defines the total score (Lv et al., 2021). In this study, a nomogram was developed based on the results of the multivariable logistic regression model to predict concentration difficulty.

### Statistical methods

Data analyses were performed using R software (4.1.3, http://www.Rproject.org) and Python (version 3.12.2, https://www.python.org). Descriptive statistics were used to characterize the participants, and Chi-squared tests were used to analyze categorical variables, expressed as frequency (%). A *p*-value of <0.05 was considered statistically significant.

## Results

### Characteristics of participants

A total of 2,146 participants were included in the analysis. Table 1 presents the descriptive characteristics of the study population. Approximately, 9.8% (*N* = 211) of participants had concentration difficulty while 90.2% (*N* = 1935) had no concentration difficulty. Further, based on the income criteria, 36.3% (*N* = 780), 14.2% (*N* = 304), and 49.5% (*N* = 1,062) had low, moderate, and high income, respectively. Among the participants, 63.7% (*N* = 1,366) had no hypertension, while 36.3% (*N* = 780) had hypertension. Moreover, 4.3% (*N* = 93), 3.7% (*N* = 79), 4.6% (*N* = 99), and 3.7% (*N* = 79) adults had a history of coronary heart diseases, stroke, liver disease, and kidney disease, respectively, with a statistical significance of *p* of <0.05.

**Table 1:** General characteristics of participants.

Concentration	No	Yes	*p*-value
*N*	1935	211	–
Sex	–	–	0.934
Male	932 (48.2%)	101 (47.9%)	–
Female	1,003 (51.8%)	110 (52.1%)	–
Age (years)	49.1 ± 17.3	53.4 ± 16.9	<0.001
Income criteria	–	–	<0.001
Low income	658 (34.0%)	122 (57.8%)	–
Median income	269 (13.9%)	35 (16.6%)	–
High income	1,008 (52.1%)	54 (25.6%)	–
BMI (kg/m^2^)	29.3 ± 7.0	31.0 ± 7.1	0.001
Hypertension	–	–	<0.001
No	1,258 (65.0%)	108 (51.2%)	–
Yes	677 (35.0%)	103 (48.8%)	–
Smoking status	–	–	<0.001
No	1,104 (57.1%)	90 (42.7%)	–
Yes	831 (42.9%)	121 (57.3%)	–
Anxiety	–	–	<0.001
Daily	219 (11.3%)	101 (47.9%)	–
Weekly	275 (14.2%)	45 (21.3%)	–
Monthly	255 (13.2%)	25 (11.8%)	–
A few times a year	709 (36.6%)	30 (14.2%)	–
Never	477 (24.7%)	10 (4.7%)	–
Depression	–	–	<0.001
Daily	45 (2.3%)	64 (30.3%)	–
Weekly	88 (4.5%)	48 (22.7%)	–
Monthly	156 (8.1%)	32 (15.2%)	–
A few times a year	646 (33.4%)	48 (22.7%)	–
Never	1,000 (51.7%)	19 (9.0%)	–
Liver disease	–	–	<0.001
No	1860 (96.1%)	187 (88.6%)	–
Yes	75 (3.9%)	24 (11.4%)	–
Stroke	–	–	<0.001
No	1877 (97.0%)	190 (90.0%)	–
Yes	58 (3.0%)	21 (10.0%)	–
Coronary heart disease	–	–	<0.001
No	1861 (96.2%)	192 (91.0%)	–
Yes	74 (3.8%)	19 (9.0%)	–
Kidney disease	–	–	<0.001
No	1876 (97.0%)	191 (90.5%)	–
Yes	59 (3.0%)	20 (9.5%)	–
Phosphorus (mmol/L)	1.2 ± 0.2	1.2 ± 0.2	0.756
Triglycerides (mmol/L)	1.4 ± 1.3	1.6 ± 0.9	0.090
Uric acid (μmol/L)	324.1 ± 85.4	322.8 ± 86.1	0.835
Sodium (mmol/L)	138.7 ± 2.0	138.6 ± 2.3	0.719
Potassium (mmol/L)	4.0 ± 0.3	4.0 ± 0.4	0.011
Iron (μmol/L)	15.3 ± 6.3	15.1 ± 6.4	0.686
Glucose (mmol/L)	5.8 ± 2.0	6.5 ± 3.2	<0.001
Chloride (mmol/L)	103.3 ± 2.9	103.6 ± 3.2	0.117
Cholesterol (mmol/L)	5.0 ± 1.1	5.0 ± 1.2	0.844
Calcium (mmol/L)	2.3 ± 0.1	2.3 ± 0.1	0.475
Sleep duration (hours)	7.6 ± 1.5	8.0 ± 2.0	0.003
Insulin (μmol/L)	84.6 ± 123.0	92.1 ± 80.0	0.383

BMI, body mass index.

### Variable selection

In LASSO algorithm, the optimal alpha parameter was 0.002. The top 14 appropriate variables included in this study are sex, age, income, BMI, sleep duration, stroke, kidney disease, liver disease, anxiety, depression, cholesterol, chloride, glucose, and sodium.

### Comparison of models

**Figure 3:**
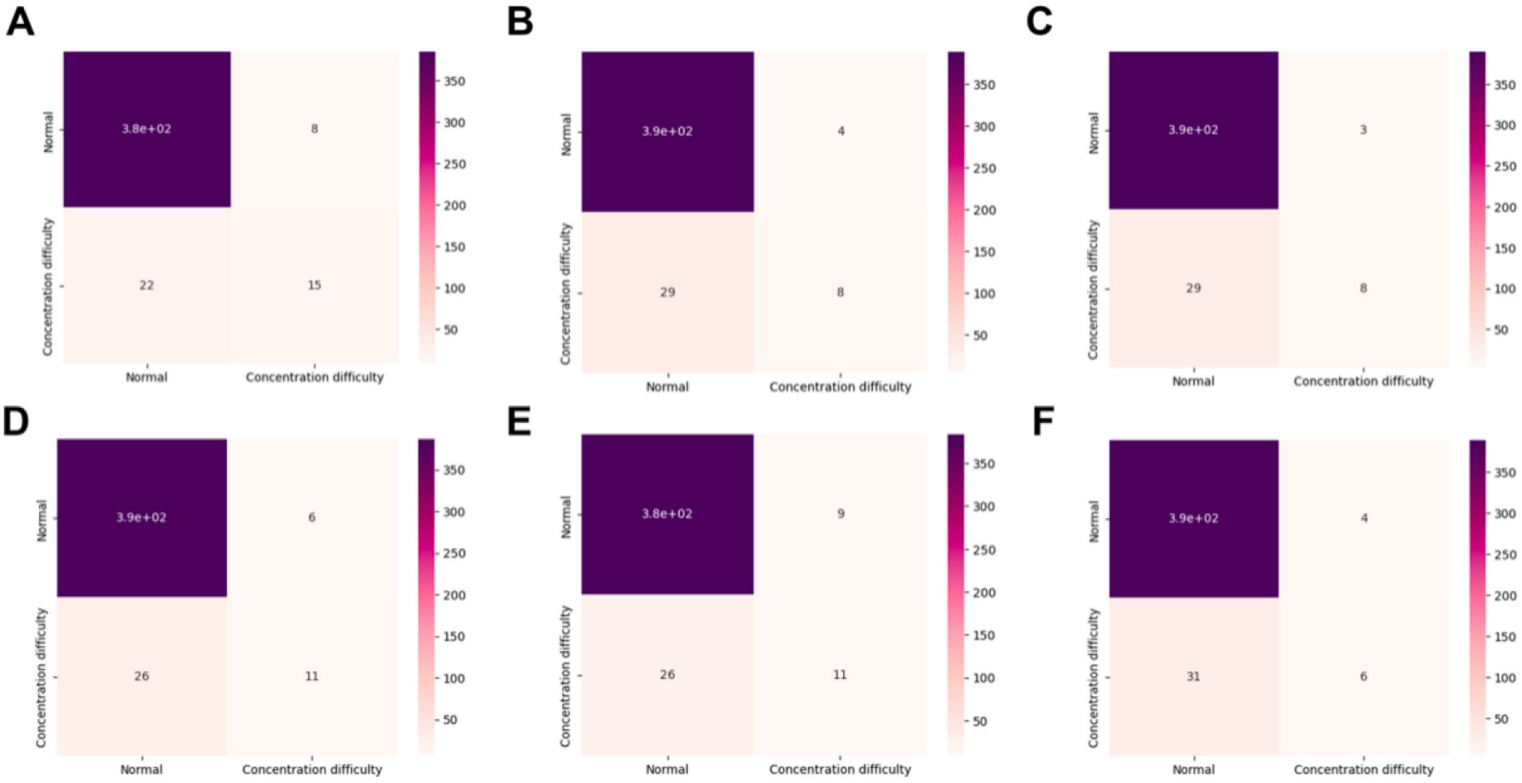
The confusion matrix for six models in the internal validation. **(A)** Logistic regression; **(B)** ExtraTrees classifier; **(C)** Bagging classifier; **(D)** Gradient boosting; **(E)** XGBoost; **(F)** RF. XGBoost, extreme gradient boosting; RF, random forest.

In this study, 5-fold cross-validation in combination with grid search was employed to determine the optimal regularization parameters for each model in the internal cohort. A confusion matrix was used to calculate various statistical metrics, including accuracy, sensitivity, specificity, positive and negative predictivity, and F1 score, as well as to evaluate the performance of each model (Guesné et al., 2024). Confusion matrices were constructed for six models in the internal validation sets to evaluate the performance of the models (Figure 3).

**Figure 4:**
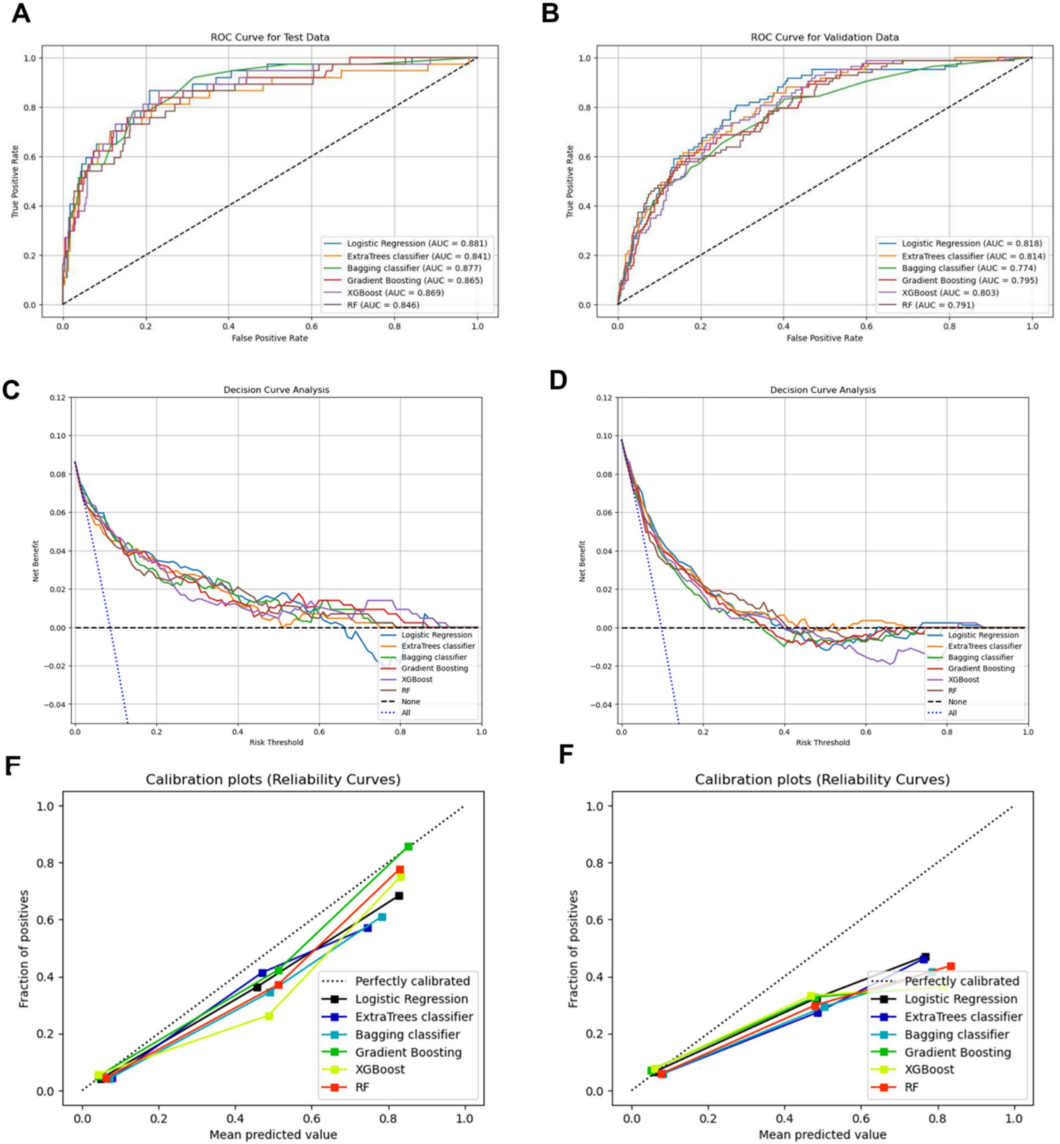
The AUC, DCA, and calibration curve of each model in the internal and external validation cohort. **(A,C,E)** Internal validation sets; **(B,D,F)** External validation sets. AUC, area under characteristic curve; DCA, decision curve analysis; XGBoost, extreme gradient boosting; RF, random forest.

As shown in Figure 4 and Table 2, logistic regression demonstrated the highest predictive performance, with an AUC curve of 0.881 in the internal validation cohort and an AUC curve of 0.818 in the external validation cohort (Figures 4A,B). Table 2 further shows that logistic regression achieved the highest accuracy (0.930) in the internal validation sets when identifying concentration difficulty. In addition, logistic regression had higher recall score (0.405) and F1 score (0.500) compared with other models (Table 2).

**Table 2:** The performance of the six prediction models in the internal validation set.

Models	Accuracy	AUC	Precision	Specificity	Recall	F1
Logistic regression	0.930	0.881	0.652	0.980	0.405	0.500
ExtraTrees classifier	0.923	0.841	0.667	0.990	0.216	0.327
Bagging	0.926	0.877	0.727	0.992	0.216	0.333
Gradient boosting classifier	0.926	0.865	0.647	0.985	0.297	0.407
XGBoost	0.919	0.869	0.550	0.977	0.297	0.386
RF	0.919	0.846	0.600	0.990	0.162	0.255

AUC, area under characteristic curve; XGBoost, extreme gradient boosting; RF, random forest; F1, the harmonic means of precision and recall.

Considering the significance of overcoming the limitations of traditional statistical metrics, DCA was employed to evaluate the clinical utility of each ML model (Zheng et al., 2023). Figure 4 illustrates the net benefit of each model along with the threshold probability. The results revealed that the net benefit of six ML algorithms was not significantly different in internal validation sets. With the risk thresholds ranging between 0.20 and 0.30, logistic regression exhibited the greatest net benefit (Figure 4C). Figure 4D depicts the net benefit curves of each model in the external validation cohort. Among the risk thresholds ranging from 0.20 to 0.30, RF demonstrates the highest net benefit value (Figure 4C).

Figures 4E,F present the calibration curve of each model in the internal and external validation cohort, respectively. Gradient Boosting exhibited superior calibration in the internal validation sets, whereas logistic regression achieved better calibration in the external validation sets (Figures 4E,F).

### Construction and evaluation of nomogram

**Figure 5:**
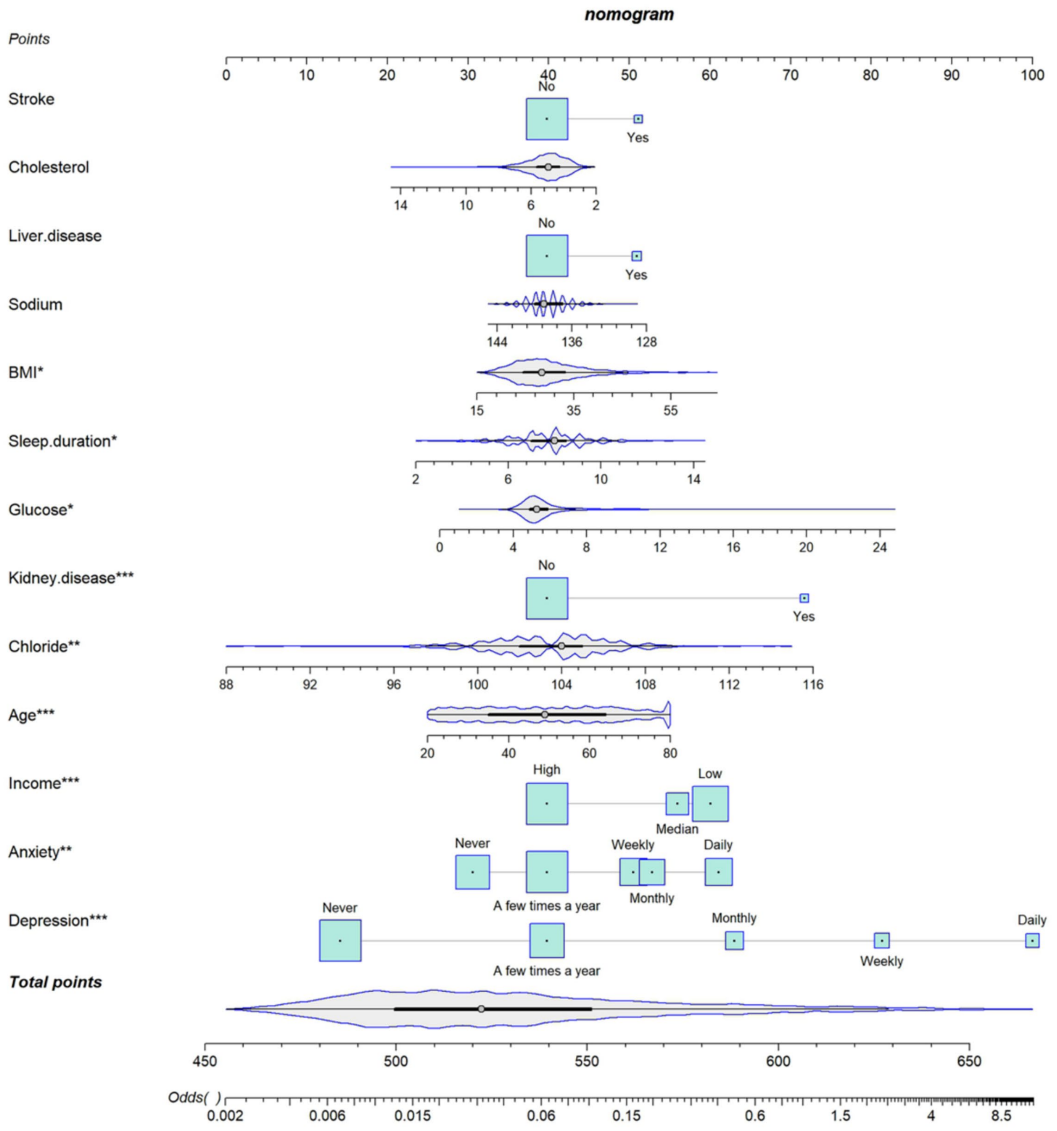
Nomogram for predicting the risk of developing concentration difficulties. BMI, body mass index.

Given the superior clinical predictive performance of Logistic Regression, a nomogram was developed by incorporating 14 key risk variables to validate concentration difficulty. The nanogram showed that daily depression corresponded to the highest risk score (100 points), followed by glucose (82 points) and chloride (75 points). For each independent risk factor, the individual score can be determined using the topmost line of the scale; then, the total score can be calculated using the lower total point scale. Clinical practitioners can evaluate the probability of attention difficulty by identifying each patient’s characteristic on the corresponding axis, awarding points, and adding them to obtain the total score. Higher total scores indicate a higher probability of concentration difficulty (Figure 5).

## Discussion

In this study, an ML model was developed to investigate the key features of the model for predicting risk factors associated with concentration difficulties using nationally representative samples from the NHANES database among adults from the United States. Approximately 14 important features were selected based on the LASSO regression, and six machine learning algorithms were employed for risk prediction. The results demonstrated that Logistic Regression exhibited the best clinical predictive value in both the internal and external validation sets, with an AUC of 0.881 and 0.818, respectively. Our findings revealed that the Logistic Regression model showed great potential in identifying the risk of concentration problems.

The results also revealed that Logistic Regression achieved higher accuracy (0.930) than other models and also exhibited the highest recall value (0.405) and F1 score (0.500). According to the DCA curve, all ML methods had a large net interest in the internal validation cohort. Of all ML methods, the Logistic Regression exhibited the highest net interest when the threshold probability varied between 0.2 and 0.3. The DCA curve exhibited that the RF model outperforms other models in the external validation cohort, indicating that RF had greater net benefit than other strategies. However, the AUC score (0.846) was comparatively lower than that in other models, and it exhibited the lowest recall value (0.162) and F1 score (0.255). Furthermore, the calibration plots revealed that the Gradient Boosting classifier exhibited superior calibration in the internal validation cohort, whereas the Logistic Regression demonstrated better calibration in the external validation cohort. These findings indicate that Logistic Regression and Gradient Boosting achieved strong agreement between the ideal and observed events in the internal and external validation cohorts, respectively. Overall, Logistic Regression surpasses the performance of other models, offering decision-making support for diagnosing attention disorders and guiding treatment interventions.

Similarly, other studies have reported similar results, indicating that Logistic Regression outperformed other algorithms. For example, Song et al. discovered that Logistic Regression exhibited an advanced performance when compared to other algorithms in predicting postoperative delirium (POD) in elderly patients, with an AUC of 0.783 (Tiwari et al., 2023). Fu et al. showed that the Logistic Regression method demonstrated superior effect in diagnosing intracranial infection, with the highest AUC value (0.847) and accuracy (0.869) (Fu et al., 2022). These studies demonstrated that Logistic Regression is a good choice for modeling as it has powerful function of handling high-dimensional spatial data effectively.

The results of this study support the previously known features associated with concentration difficulties, such as age, depression, stroke, kidney disease, liver disease, and anxiety (Paelecke-Habermann et al., 2005; Xu et al., 2022; Viggiano et al., 2020; Weissenborn et al., 2005; Najmi et al., 2012). Among them, depression is the most important feature for predicting concentration disorders. The findings also showed a positive correlation between depression and impaired attention. Another research revealed that patients with ADHD had a 20% lower rate of depression after receiving treatment when compared with the untreated group (Chang et al., 2016). Similarly, compared with healthy individuals, patients with MDD had lower levels of brain-derived neurotrophic factor (BDNF) and poorer performance in attention (Teng et al., 2021). Except for depression, a previous study also considered anxiety as a common diagnostic criterion for concentration difficulty (Hao et al., 2025). It is known that the elderly have a significant tendency to attention disorders. Nevertheless, impairments in attention can also be detected in individuals of different age groups, including those with epilepsy (Brissart et al., 2019). Commodari and Guarnera (2008) performed an age-related attentive efficiency and found that subjects aged 55–59 outperformed subjects aged 60–65. Compared to Commodari’s study, this study performed a survey on individuals aged more than 20, which is a more comprehensive way. Fisk et al. (2002) demonstrated that stroke survivors are more likely to have attention deficits than those without stroke. Even subcortical “mini-strokes” may exhibit significant difficulties with attention (Soleimani et al., 2023). Both liver diseases and kidney diseases have significant effects in attention. For example, a review reported by Pepin et al. demonstrated significant improvements in attention in patients with chronic kidney disease (CKD) after kidney transplantation (Pépin et al., 2021). In addition, there have been various reports of cognitive decline in patients with hepatic encephalopathy or renal encephalopathy. Impairment in attention is one of the characteristics of patients with minimal hepatic encephalopathy (Bajaj et al., 2008). The findings indicated that the risk factors we identified as being associated with concentration difficulty were both reliable and practicable.

In this study, unexpected features that are easily ignored in clinical practice were also identified, such as BMI, glucose, and chloride. For example, van Mil et al. (2015) found that children with a higher birth weight exhibited fewer attention issues, particularly when their birth weight was below 3.6 kg. However, few studies have examined the relationship between BMI and adult concentration. Although previous studies have revealed that both type 1 and type 2 diabetes contribute to attention disorders, the correlation between serum glucose levels and an impairment in attention has been rarely documented to date. The findings also revealed that the serum glucose of the participants may contribute to attention issues. In addition, to our knowledge, this is the first study to identify serum chloride as a risk factor for attention difficulties. Nevertheless, the fundamental biological process behind the decrease in attention still requires additional investigation.

The ML models developed in this study accurately assessed attention disorders, which may facilitate medical institutions in adopting intervention strategies to reduce associated risks. In addition, these models can be used in clinical consultations, particularly in remote areas where detailed evaluation is not possible. Moreover, the nomogram revealed important risk characteristics associated with attention disorders. Clinicians can use this tool to evaluate the risk of attention difficulty in individuals, thereby enabling more accurate identification and prioritization on effective treatment strategies.

This study had several strengths. First, although ML has been widely applied in predicting concentration difficulties, a majority of previous studies have focused on children. Second, this study is the first to apply ML algorithms to construct six models for the prediction of concentration difficulties in adults. Third, to improve the performance of the model, a cross-validated grid search was employed to evaluate the hyperparameter values for each algorithm. Finally, the performance of the prediction models was assessed using an external validation cohort.

This study had several limitations. First, although questionnaires have been commonly used to assess attention disorders in previous studies, they remain subjective and susceptible to interference from several factors. Second, since relevant data were acquired from the United States, the performance of the proposed model remained unclear in other populations, such as Chinese. In our future research, we will focus on validating the model across diverse populations.

## Conclusion

The Logistic Regression model achieved the strongest predictive performance, with the highest AUCs in the validation sets [internal (0.881) and external (0.818)]. Logistic Regression also provided the largest net benefits in the internal cohort. Depression was identified as the most critical predictor in the nomogram analysis.

## Data Availability

The raw data supporting the conclusions of this article will be made available by the authors, without undue reservation.
